# Preventing Transfusion-Transmitted Babesiosis

**DOI:** 10.3390/pathogens10091176

**Published:** 2021-09-13

**Authors:** Evan M. Bloch, Peter J. Krause, Laura Tonnetti

**Affiliations:** 1 Division of Transfusion Medicine, Department of Pathology, Johns Hopkins University, Baltimore, MD 21287, USA; 2 Department of Epidemiology of Microbial Diseases, Yale School of Public Health, New Haven, CT 06520, USA; peter.krause@yale.edu; 3 Scientific Affairs, American Red Cross, Holland Laboratories, Rockville, MD 21287, USA; Laura.Tonnetti@redcross.org

**Keywords:** *Babesia*, blood transfusion, prevention, screening, babesiosis

## Abstract

*Babesia* are tick-borne intra-erythrocytic parasites and the causative agents of babesiosis. *Babesia*, which are readily transfusion transmissible, gained recognition as a major risk to the blood supply, particularly in the United States (US), where *Babesia microti* is endemic. Many of those infected with *Babesia* remain asymptomatic and parasitemia may persist for months or even years following infection, such that seemingly healthy blood donors are unaware of their infection. By contrast, transfusion recipients are at high risk of severe babesiosis, accounting for the high morbidity and mortality (~19%) observed in transfusion-transmitted babesiosis (TTB). An increase in cases of tick-borne babesiosis and TTB prompted over a decade-long investment in blood donor surveillance, research, and assay development to quantify and contend with TTB. This culminated in the adoption of regional blood donor testing in the US. We describe the evolution of the response to TTB in the US and offer some insight into the risk of TTB in other countries. Not only has this response advanced blood safety, it has accelerated the development of novel serological and molecular assays that may be applied broadly, affording insight into the global epidemiology and immunopathogenesis of human babesiosis.

## 1. Introduction

*Babesia* are tick-borne apicomplexan parasites and the causative pathogens of the clinical illness, babesiosis. Over 100 species of *Babesia* infect a wide array of vertebrates, yet only six species have been implicated in human infections, of which *Babesia microti* is overwhelmingly predominant [1]. While *B. microti* has been reported frequently from the northeastern and northern midwestern United States (US), cases of babesiosis have been described globally [2]. Findings from *Babesia* surveillance and clinical case reporting suggest a significant increase in *B. microti* incidence in the United States (US) over the past two decades [3]. Factors that have been postulated for the emergence of *Babesia* include an increase in the deer population that amplifies the number of ticks, an increase in the human population, and building homes in tick infested areas [3,4,5]. *Babesia* was historically under-investigated, whereby greater attention (i.e., awareness) following its becoming a notifiable disease in many US states in 2011 likely contributed to the observed increase in cases.

*Babesia* are transmissible through blood transfusion [6]. The increase in reported cases of naturally acquired and transfusion-transmitted babesiosis (TTB) in the US drew the attention of the blood banking community, thus prompting over a decade of donor surveillance studies, along with the development of laboratory-based diagnostic and donor screening strategies to contend with TTB [7,8]. This culminated in 2019 with the publication of nonbinding recommendations from the US Food and Drug Administration (FDA) in favor of regional blood donor screening for *Babesia* in the US using an approved molecular assay [9]. Prior to the adoption of laboratory-based screening, *B. microti* was a leading infectious risk to the US blood supply. The risk of TTB in the US is now low as a result of routine testing for *Babesia* [10]. We describe the evolution of the response to TTB in the US as a means to contextualize the risk of *Babesia* in general with a view to guide future research efforts.

## 2. Epidemiology: Geographic Distribution, Seasonality, and Transmissibility

*Babesia* species have different geographic distributions. Cases of *B. microti* have been reported widely, notably in the northeastern and upper midwestern US, but also in other countries [11,12,13,14,15]. *Babesia duncani* occurs in the far western US [16]. *Babesia venatorum* and *Babesia crassa*-like agent have been reported in Europe and northeastern China [17,18,19]. *Babesia divergens*/*Babesia divergens*-like agents has been reported in Europe [17] and the United States [20]. *Babesia motasi*-like agent has been implicated in human cases in Korea [21]. 

### 2.1. Blood Donor Surveillance in the US

Beginning in the late 1990’s, a series of surveillance studies were conducted to determine the seroprevalence of *Babesia* (specifically *B. microti*), as well as rates of parasitemia (using molecular positivity as a surrogate of active infection) specific to the blood donor population (Table 1). In one of the earliest studies, blood donors (*n* = 3490) in endemic and nonendemic areas of Connecticut were evaluated for *B. microti* [22]. In this study, 30 (0.9%) donors were confirmed positive for antibodies against *B. microti*; over half (10/19) of seropositive donors who were subsequently tested by PCR were shown to be positive [22]. In another study, about a fifth (21%) of 84 seropositive blood donors (IFA titers ≥ 64), who were followed for up to three years in Connecticut and Massachusetts, were found to be parasitemic [23]. Over the course of follow-up, protracted low-level parasitemia was variably and intermittently detectable. 

### 2.2. TTB in the US

*Babesia* are intraerythrocytic parasites and are readily transmissible through transfusion of any product containing red blood cells. TTB has been reported following transfusion of whole blood, packed red blood cells (RBCs), and even frozen RBCs [6]. Confirmed cases of TTB have not been ascribed to transfusion of apheresis platelets and acellular blood products such as plasma and cryoprecipitate [6]. Rare cases of TTB have been reported after transfusion of whole blood-derived platelets [6]. This may have been due to contamination of red cells and/or the presence of extraerythrocytic parasites [34]. The minimum infectious dose of *B. microti* that can cause TTB is low (10–100 parasites), based on murine models [35] (Table 2). TTB following transfusion of pediatric red cell aliquots and whole blood-derived platelets suggests that infectivity is high. 

To date, over 250 cases of TTB have been reported in the US, almost all (98%) of which were caused by *B. microti*. There are three reports of TTB due to *B. duncani* and one due to *B. divergens*-like parasites [20,41]. The risk of TTB is more widespread in the US than that associated with tick-borne transmission of parasites. Blood is often transfused far from where it is collected. It is not uncommon for blood products to cross state lines, where distribution is driven by clinical need, disproportionately being drawn toward major urban centers. In addition, residents from nonendemic areas may become asymptomatically infected during travel to endemic areas, return home, and donate blood [42]. *B. microti* can persist for long periods of time, even after standard antimicrobial therapy, whereby asymptomatic individuals may donate long after becoming infected [42,43]. These factors have accounted for cases of TTB in nonendemic states [44,45]. 

Natural acquisition of *Babesia* is predominantly seasonal, with peak incidence spanning late spring to early fall, following the life cycle of the tick vector. By contrast, cases of TTB are not strictly confined to peak periods of vector-borne transmission, although they still have a similar time distribution pattern as tick-borne disease, having been reported throughout the year [6,46]. Prolonged storage of blood components enables transfusion of parasitemic blood long after donor acquisition of infection and expands transmission time to include the entire calendar year [8,43]. In addition, the incubation period for development of symptoms after transfusion is as long as six months [6]. Furthermore, donor surveillance studies and prospective screening have also identified parasitemic donations (i.e., positive nucleic acid test) throughout the year, although positive donations still tend to occur from June to October [33]. 

## 3. The Risk of Transfusion-Transmitted Babesiosis outside of the US 

To date, cases of TTB have been almost exclusively described in the US, with rare exceptions of reports in Japan and Canada (Table 3) [47]. Although it is well established that *Babesia* is globally ubiquitous, few studies have been undertaken to quantify risk of TTB outside of the US. 

A seroprevalence study was undertaken of Tyrolean blood donors (*n* = 988): 2.1% were IgG-positive against the *B. divergens* complex and 0.6% were seropositive for *B. microti* [48]. While both species are causes of human infections, *B. divergens* has not been found to be transmitted through blood transfusion. 

Canada has plausible risk given its proximity to endemic US states, as well as previously described autochthonous cases. In one study, passive surveillance was utilized to guide follow-up active surveillance and intervention [49]. Specifically, ~12,000 ticks that had been submitted by the public were tested for evidence of *Babesia* infection. Fourteen were found to be *B. microti*-positive, 10 of which originated in Manitoba. This guided selection of regions for active surveillance (2009–2014) using tick drag sampling. The ticks were tested by PCR: 6/361 (1.7%) were positive in Manitoba and 3/641 (0.5%) were positive in Quebec. None were positive from other sites. Blood donations (July and December, 2013) at selected sites near endemic US regions were tested for antibodies to *B. microti*. A donor questionnaire was used to enquire about travel-related risk and possible tick exposure. A total of 13,993/ 26,260 (53%) donors were tested, none of whom were found to have antibodies to *B. microti.* Further, almost half (47%) reported having visited forested areas in Canada and 41% had traveled to the US. During a more extensive study performed in 2018, over 50,000 donations that had been collected near the US border were tested for *Babesia* nucleic acid by transcription-mediated amplification (TMA). In addition, a subset of 14,758 TMA-nonreactive samples was also screened for *B. microti* antibodies. The study identified one TMA-reactive donation that had been collected in Winnipeg, Manitoba, the only region in Canada where autochthonous infections have been reported, and four antibody-positive donations in the TMA-negative group [51]. Collectively, these findings suggest that the risk of TTB is low in Canada and that a risk-based deferral for *Babesia* is not needed at the moment.

A study was conducted in blood donors in China [50]. Again, there is a plausible regional risk given prior reports of human babesiosis in China, as well as in Mongolia, Korea, and Japan [14,54,55,56,57,58]. A total of 1000 donor samples representing 888 whole blood and 112 platelet donations that had been collected in Heilongjiang province were evaluated by IFA against *B. microti:* 13/1000 (1.3%) were seroreactive. 

In Australia, a fatal case of autochthonous babesiosis due to *B. microti* raised concern pertaining to the national blood supply [11,59]. A total of 7000 donations were tested for anti-*B. microti* IgG by IFA [52]. Initial reactive samples were subjected to *B. microti* IgG and IgM (immunoblot), as well as PCR. Five donors were initially reactive by IFA, none of whom were confirmed during repeat testing. All were PCR-negative. In addition, clinically suspected cases of babesiosis (*n* = 29) were also evaluated; none were *B. microti* IgG, IgM, or DNA positive. 

## 4. Clinical Presentation

Clinically, about a fifth of *Babesia* infections in adult immunocompetent hosts are subclinical or manifest as mild flu-like illnesses that are not diagnosed and often clear without treatment [60]. Most patients experience a mild to moderate febrile illness that typically consists of fatigue, headache, chills, and sweats. However, selected patient subsets are at high risk of severe disease with complications. The latter include hemolytic anemia; cardiorespiratory, renal, and/or liver failure; disseminated intravascular coagulopathy; and death [2]. Transfusion recipients harbor many of the risk factors for severe or even fatal babesiosis, such as advanced age, comorbid cardiac or pulmonary disease, immunodeficiency due to asplenia, cancer, HIV/AIDS, or sickle cell disease [2]. This helps to explain the severity of illness and high fatality rate (~19%) associated with transfusion-transmitted babesiosis (TTB) [1,6,60]. Indeed, variability in reported fatality rates from babesiosis, in general, largely reflects a difference in clinical penetrance that is governed by the immune status of the host [61,62,63]. Importantly, transfusion of red blood cells and whole blood is indicated for the treatment of severe, decompensated anemia. Therefore, parasite-induced hemolysis that might otherwise be tolerated in the immunocompetent individual can have dire consequences in the transfusion recipient.

## 5. Prevention Strategies 

### 5.1. Risk-Based Deferral

Historically, prevention of TTB has relied on donor selection (Table 4). Individuals who reported a history of babesiosis were permanently deferred from blood donation. This proved suboptimal, as evidenced by the number of cases of TTB that escaped detection using this approach. There are a number of reasons why this approach was problematic. For one, *Babesia* are able to persist chronically in donors without apparent adverse effects [42]. Even when clinically overt, the symptoms of babesiosis in immunocompetent adults are nonspecific. Risk factors for tick exposure are also nonspecific (e.g., outdoor activities, residence in highly endemic states), offering little diagnostic utility [32]. Vector (i.e., tick)-borne transmission is seasonal, largely aligning with the tick life cycle, whereby most infections occur late spring to early fall [2]. By contrast, cases of TTB are less prone to seasonality, given that blood can be stored for prolonged periods. Further, persistent, asymptomatic infection is well described, in some cases being detectable for more than two years following infection [42,43]. 

A history of tick bites is also a poor predictor of infection. Recall of tick bite is unreliable. One study observed no significant difference in *Babesia* seroprevalence between those who reported tick bites as compared to those who did not [64]. The investigators postulated that those who report tick exposure are the same group who take precautions against tick bites. Importantly, a high proportion of infections are ascribed to the bites from nymphs rather than adult ticks. Nymphal ticks are the size of poppy seeds, rendering them highly inconspicuous. 

### 5.2. Laboratory-Based Methods for Donor Screening 

Laboratory testing is necessary for any meaningful donor screening intervention. Laboratory approaches in routine use for clinical diagnosis of babesiosis (e.g., microscopy of peripheral blood smears and manual indirect fluorescent antibody [IFA] testing) are not suitable for donor screening. Microscopy is neither scalable nor sufficiently sensitive or specific to detect the low level of parasitemia that is often encountered in blood donors. Manual IFA testing is not amenable to high-throughput screening. Molecular testing for *Babesia* is a more suitable approach for blood donor screening but poses novel challenges. *Babesia*—unlike the major transfusion-transmitted viruses— is primarily red-cell-based, thereby requiring additional processing steps for optimal sensitivity of detection. Given the large numbers of donors, automation is critical. Therefore, a process needed to be devised to better access the target parasites in the infected red blood cells. 

### 5.3. Serological Testing 

The initial approach for evaluating *Babesia* in the blood donor population was focused on serology (i.e., antibody capture)—specifically of anti-*B. microti* antibodies— in endemic areas. Experimental research assays were developed for the detection of *B. microti*. One approach used an enzyme immunoassay (EIA) (i.e., targeting the recombinant protein BMN-17 and MN-10) [36]; the other employed a semi-automated IFA test [22]. Although less labor-intensive, the EIA assay showed poor specificity as compared to the semi-automated IFA test. By contrast, IFA testing is sensitive and specific and is still used today to supplement positive nucleic acid test results. The semi-automated version of the IFA test, the arrayed fluorescent immunoassay (AFIA), was applied successfully in a series of donor surveillance studies [8,10]. The combination of AFIA and real-time PCR were the first tests to receive FDA licensure for screening of blood donations, but have since been discontinued for blood screening by the manufacturer [65]. 

Another antibody test, an enzyme-linked immunoassay (ELISA), was developed to detect antibodies against *B. microti*. The assay employed four immunodominant peptides from the BMN1 family that had been shown to be immunodominant and highly specific to *B. microti* [36]. The assay was capable of detecting both IgM and IgG against *B. microti* [29]. In a pilot study, 15,000 blood donor samples from high-risk, low-risk, and nonendemic areas of New York State (5,000 each) were tested. Rates of reactivity following application of a revised cutoff were 0.92%, (46/5000), 0.54% (27/5000), and 0.16% (8/5000), respectively [29]. ELISA repeat-reactive samples were also tested by IFA with a concordance rate of 99.34%. Although the ELISA was evaluated in a formal IND (investigational new drug) trial, which was a preliminary step along the regulatory pathway to licensure, the assay was never licensed and is no longer in use. 

### 5.4. Molecular Testing

Molecular testing better detects active infection/parasitemia than antibody testing. This is important because active infection rather than *Babesia* exposure alone (i.e., antibodies), is required for transmission by blood transfusion. Nevertheless, mitigation strategies for blood donors focused initially on serological methods. Molecular assays (i.e., nucleic acid testing or NAT) have been used since ~1999 to detect the major transfusion-transmissible viruses (e.g., HIV, hepatitis B, and hepatitis C viruses) [66]. Those agents are detectable in plasma. By contrast, *Babesia* are primarily intraerythrocytic, requiring additional processing of whole blood to ensure adequate target capture. 

A variety of PCR research assays, from nested to real-time, have been developed using the 18S ribosomal RNA gene of *B. microti* as a target and used to determine parasitemia in antibody-positive blood donations during surveillance studies [27,67]. In most cases, these assays have been shown to be sensitive and specific; however, the methodologies used to access the red cell compartment represented a limiting factor for the sensitivity of these assays for blood donor screening. In addition, hemoglobin is also a known inhibitor of PCR [68]. The first real-time PCR assays for donor screening utilized an automated membrane-based isolation system (Taigen Bioscience) and had a limit of detection of 66 piroplasms per mL [8]. Later, larger manufacturers such as Grifols Diagnostics and Roche developed assays, and ultimately obtained FDA licensure [69,70]. These assays are exquisitely sensitive and specific, attaining limits of detection for *Babesia* as low as 2–3 parasites/mL [66]. Both assays can detect ribosomal DNA or RNA of four major species of *Babesia* that infect humans (*B. microti*, *B. divergens*, *B. duncani*, and *B. venatorum*) [38]. The assays can be performed on an automated platform and in pools of 6 (Cobas Babesia, Roche Diagnostics) to 16 samples (Procleix Babesia assay, Grifols diagnostic solutions), allowing for the screening of large numbers of donations. One of the two assays in current use (Cobas Babesia, Roche Diagnostics) employs proprietary whole blood collection tubes containing lysing agents [38]. 

## 6. Economic Impact

The cost implications of donor screening have been assessed in three studies undertaken by different groups. The first study examined four different testing strategies as applied to endemic areas: universal antibody screening, universal molecular screening, universal combined testing (antibody/molecular), and recipient-risk-targeted combined (antibody/molecular) testing [71]. The strategies were compared to the then-current standard practice of using a questionnaire. The authors concluded that use of a questionnaire was most wasteful, followed by a risk-targeted combined approach. Universal molecular screening would incur an incremental cost-effectiveness ratio (ICER) of $26,000 to $44,000/ quality adjusted life year (QALY) and would serve to prevent 24 to 31 TTB cases/100,000 units transfused, incurring no wastage. The combined approach would be more effective, albeit at a higher cost. By contrast, antibody-based screening was lower in cost, yet was less effective and incurred higher wastage than the molecular options. 

The second analysis evaluated the cost utility of a similar repertoire of screening approaches in endemic areas [72]. The results were substantially different. For one, the ICER for combined testing as compared to antibody screening was in excess of $8.7 million, preventing 3.6 cases of TTB per 100,000 units transfused. Universal endemic antibody screening was projected to prevent 3.39 cases of TTB at an ICER of $760,000/QALY when compared to the recipient-risk-targeted strategy. The authors concluded that antibody was the most cost-effective strategy when applying the threshold of cost effectiveness specific to transfusion safety initiatives in the US, i.e., $1 million/QALY. 

The third study examined the cost-utility of different screening strategies, both by mode of testing (IFA, ELISA, PCR), as well as extent of geographic inclusion [73]. The authors concluded that even a strategy that was to be confined to highly endemic states would likely exceed the implicit threshold for cost-effectiveness of $1 million per QALY. 

## 7. US Policy 

Babesiosis has long been recognized as posing a risk to the US blood supply [9,74]. However, availability of validated tests that were of sufficient level of performance for donor screening, impeded rapid adoption of preventive strategies [75]. In 2019, the US FDA published their recommendations, thus supporting regional molecular screening of blood donors in 14 states and Washington DC using any of the approved assays [9]. Over 95% of all cases of TTB and 99% of clinical cases of babesiosis have occurred in the selected locations. The recommendations also allowed for pathogen reduction (PR) as an alternative to laboratory testing. At the time of this writing, at least one PR technology had been FDA approved for use in plasma and platelets. Of note, neither plasma nor apheresis platelets pose significant—if any—risk of transfusion transmission. A history or babesiosis or a positive test for *Babesia* previously led to permanent deferral from blood donation. Under the new guidance, donor re-entry is allowable after 2 years in the event that the donor has not had a positive test result for *Babesia* during the interval, remains negative by requalification using one of the licensed *Babesia* NAT assays, and meets all other eligibility criteria for blood donation [9]. 

## 8. Discussion

Successful strategies to reduce the risk of the major transfusion-transmitted viruses (e.g., HIV, hepatitis B, and C viruses) have rendered blood transfusion remarkably safe, at least in the US and other high-income countries [66,76]. These successful strategies have contributed to the investigation of risk posed by other pathogens (e.g., *Babesia*) and classes of pathogens (e.g., bacteria) to the blood supply. Implementation of donor screening for *Babesia* in the US has been a success, having —arguably— removed one of the last major transfusion-transmissible infections, thus serving to advance blood transfusion safety nationally. 

Nonetheless, the donor screening policy was long overdue. A potential contributing factor for the delayed development of *Babesia* blood screening assays was the evolution of *T. cruzi* screening in the US. *T. cruzi*, the causative parasite for Chagas disease, is transfusion-transmissible. The agent is endemic to Central and South America, where longstanding public health efforts coupled with serological testing of blood donors have contributed to a decline in cases [77]. Universal donor screening for Chagas disease began in the US in 2006. Following implementation, studies determined the risk in the US to be low. This prompted a revision of the policy at that time to restrict screening to first-time donor testing only. While rational in outlook, that shift in policy impaired commercial investment in testing. The downstream effect may have been the later, tepid support from the major test manufacturers—at least initially—for *Babesia* testing. Instead, the larger blood collection agencies, such as American Red Cross, Vitalant (then Blood Systems), and New York Blood Center, partnered with small businesses to develop assays. 

The path to regulatory approval and development of a screening policy for *Babesia* took almost a decade. By way of comparison, implementation of routine testing for West Nile Virus in 2003 (lauded as a major success) took less than a year from recognition of transfusion-transmitted disease [78], a timeline bettered by the later adoption of screening for Zika in 2016 within weeks [79,80,81]. Of note, Zika has yet to show any evidence of clinical effect following the rare accounts of possible transfusion transmission. Collectively, this underscores the myriad of factors and competing priorities that guide blood transfusion policy, not all of which are scientific in nature [82]. 

While there may be an element of closure on TTB in the US, *Babesia* remain global pathogens. *Babesia* species have been described in both ticks and animal populations over a wide geographic distribution spanning the Americas, Europe, Asia, Africa, and Australia [2,11,12,13,17,55,58,83]. Outside of the US, perception of risk is low and the US remains the only country to have implemented blood donor screenings [77]. Over the last two decades, only six studies (and two case reports) pertaining to TTB have originated outside of the US (Table 3). Those studies did not find comparable risk to that encountered in the US [49,52,53]. Nonetheless, surveillance is lacking, with a grossly skewed geographic sampling that remains focused on the US. One of the challenges that previously impeded surveillance was the lack of diagnostic tools that could be applied to high-throughput testing. The advent of licensed, high-performance commercial *Babesia* PCR and TMA assays should enable testing across a more diverse geography, with the caveat that implementation of molecular testing, even for research use, is challenging for low- and middle-income countries [84]. While robust molecular assays may be available, the lack of local expertise and infrastructure may still necessitate the transfer of samples to settings where equipment is available. 

## 9. Conclusions

*Babesia* are major transfusion-transmissible parasites. A concerted effort by the blood banking community has yielded effective policy and testing strategies that have been integrated into routine donation practices in the US. Nonetheless, these efforts have not been matched elsewhere and deserve greater attention from the international blood banking community. Further, the lessons learned from *Babesia* (e.g., related to sample preparation, thus enabling automated testing of an intraerythrocytic pathogen) can be applied to P*lasmodium* (malaria), a related parasite that remains a leading cause of transfusion-associated morbidity in much of the World.

## Figures and Tables

**Table 1:** Transfusion-transmitted babesiosis: blood donor surveillance and follow-up studies in the United States.

Overview	Study Design	Location (s)	Year (s)	Major Finding	Reference
Donor surveillance (research)	3490 donations (1745 each fromendemic and nonendemic areas) were tested for *B. microti* antibodies using research-based enzyme immunoassay (EIA); supplemental IFA was used to conform EIA+ samples. Selected seropositive samples were evaluated using nested PCR.	CT, USA (endemic and nonendemic areas)	1999	30/3490 (0.9%) confirmed as seropositive (*n* = 24 [1.4%] vs. 6 [0.3%] in endemic and nonendemic areas, respectively). 10/19 (53%) of 19 seropositive donors PCR+.	Leiby et al. Transfusion 2005 [22]
Donor surveillance (research)	23,304 donations from 17,465 donors were tested by IFA.	CT and MA, USA	2000–2007	267/23,304 (1.1%) seroprevalence.	Johnson et al. Transfusion 2009 [24]
Donor surveillance (research)	Cross-sectional IFA (*B. microti* IgG) testing of blood donors with PCR testing of seroreactive donors and lookback investigation.	CT, USA	1999–2005	208/17,422 (1.2%) IFA+ 26/139 (18.7%) PCR+ 8/63 recipients were IFA and/or PCR+.	Johnson et al. Transfusion 2011 [25]
Donor screening and follow-up (research)	*B. microti* IFA (titers ≥ 64) were monitored up to 3 years for parasitemia by 2 PCR methods and hamster inoculation.	CT and MA, USA	2000 to 2004	18/84 (21.4%) donors parasitemic at follow-up; 9 had >1 specimen with evidence of parasitemia. Observation of protracted, intermittent, low-level parasitemia.	Leiby et al. Transfusion 2014 [23]
Hemovigilance study	Description of donor and recipient characteristics of suspected cases of TTB reported to American Red Cross.	USA (national)	2005 to 2007	Eighteen definite or probable B. microtiinfections with 5 fatalities 4/18 (24%). Nonresident donors had a history of travel to endemic areas.	Tonnetti et al. Transfusion 2009 [26]
Donor surveillance with prospective follow-up (research)	Cross-sectional surveillance of consenting blood donors using RT-PCR and IFA (*B. microti* IgG); blood donors in VT (non or low-endemic state) used to establish specificity.	Southeast CT, USA VT, USA	2009	25/1002 (2.5%) IFA+ 3/1002 (0.3%) PCR+ (1 was IFA-negative). 1/1015 (0.1%) Vermont donors was IFA+.	Johnson et al. Transfusion 2013 [27]
Donor screening (real-time/operational)	Selective real-time donor screening with IFA and PCR units directed to neonates and pediatric sickle cell and thalassemia patients.	RI, USA	2010–2011	26/2113 (1.23%) representing 1783 blood donors were IFA+. 1 indeterminate PCR result (0.05%). No cases of TTB (vs. 7 cases of TTB out of 6500 unscreened units in targeted population using historical controls (2005–2010).	Young et al. Transfusion 2012 [7]
Investigation screening using donor sample repository	Paired samples screened by AFIA and PCR.	Nonendemic (AZ and OK), moderately endemic (MN and WI), and highly endemic (CT and MA) areas of the USA	2010 to 2011	Positivity (Seroreactivity and/or PCR+): Nonendemic: 0.025% (95% CI, 0.00–0.14%); Midendemic: 0.12% (95% CI, 0.04–0.28%); High endemic: 0.75% (95% CI, 0.53–1.03%). AFIA specificity 99.95% and 99.98% at cutoff of 1-in-64 and 1-in-128, respectively.	Moritz et al. Transfusion 2014 [28]
Validation study of EIA for blood donor screening	Retrospective testing of donor samples collected in high-risk endemic, lower-risk endemic, and nonendemic; EIA+ samples further tested by *B. microti* IFA, PCR, and peripheral blood smear examination.	Nonendemic area: AZ Lower-risk area: Manhattan and Brooklyn, NY High-risk endemic: Suffolk County, NY	2012	EIA repeat-reactive rates: Nonendemic area: 8/5000 (0.16%); Lower-risk area 27/5000 (0.54%); High-risk endemic: 46/5000 (0.92%).	Levin et al. Transfusion 2014 [29]
Donor screening (real-time/operational)	Donor screening with PCR and arrayed fluorescence immunoassay (AFIA).	CT, MA, MN, and WI, USA	2012 to 2016	700/220,749 donations screened positive, of which 15 (1 per 14,699 donations) were deemed to be window period infections (PCR+/AFIA-). Median estimated parasite load in WP donations 350 parasites/mL 3/10 (30%) WP donations infected hamsters.	Moritz et al. Transfusion 2017 [30]
Donor screening (real-time/operational)	Prospective AFIA and quantitative PCR testing of blood donors for *B. microti* DNA; assessment of parasitemia and infectivity using xeno-inoculation of hamsters. Prospective follow-up of test-reactive donors.	CT, MA, MN, and WI, USA	2012 to 2014	89,153 blood donation samples tested: 335 (0.38%) confirmed positive and 67/335 (20%) PCR-positive; 9 samples PCR+ but AFIA- (1 in 9906 screened), 27/93 (29%) reactive samples were infectious when inoculated into hamsters. At 1-year follow-up, DNA clearance had occurred in 86% of test-reactive donors but antibody seroreversion observed in only 8%.	Moritz et al. Transfusion 2016 [8]
Real time screening and donor notification	Screening blood donors with an investigational *B. microti* EIA. Repeat-reactive samples were retested by PCR, blood smear, IFA, and immunoblot assay. Findings were correlated with samples that had been collected from patients with established diagnoses of babesiosis.	NY, MN, and NM, USA, representing high endemic, moderately endemic, and nonendemic areas, respectively	2013	Rates of repeat reactivity by EIA: 38/ 13,757 (0.28%) NY; 7/4583 (0.15%) MN; 11/8363 (0.13%) NM. 9/56 EIA repeat-reactive donors positive by PCR. Assay specificity 99.93%. Sensitivity 91.1%.	Levin et al. Transfusion 2016 [31]
Donor follow-up study	Prospective evaluation of seroreactive blood donors identified during study by Levin et al. [31]. Repeat testing (PCR, IFA, EIA, and blood smear) and completion of clinical questionnaire over the course of ~12 months of follow-up after reactive donation.	NY, MN, and NM, USA representing high endemic, moderately endemic and nonendemic areas respectively	2013–2014	37/60 (61.67%) eligible seroreactive donors enrolled, of whom, 20 (54%) completed the 12-month follow-up: 15/20 (75%) were still seroreactive at follow-up. 5/9 PCR+ donors participated in follow-up study: two remained positive at final follow-up (378 and 404 days). Most seroreactive donors exhibited low-level seroreactivity that was stable or waning. Level and pattern of reactivity correlated poorly with PCR positivity.	Bloch et al. Transfusion 2016 [32]
Donor screening (real-time/operational)	Donor screening with transcription-mediated amplification (Procleix Babesia assay, Grifols Diagnostic Solutions) in in 11 endemic states; minipool and individual donor testing evaluated.	11 endemic states, Washington DC and Florida	2017–2018Extended to 2019	61/176,608 donations confirmed positive (1 in 2895 donations). Extended screening 211/496,270 (1 in 2351 donations) confirmed positive. Detection of positive donations not restricted by season. 6 positive donations identified in individual testing also detected through pooled testing. 100% specificity (no false positives).	Tonnetti et al. Transfusion 2020 [33]

AFIA—arrayed fluorescent immunoassay (AFIA); IFA—indirect fluorescent antibody; EIA—enzyme immunoassay; PCR—polymerase chain reaction; TTB—transfusion-transmitted babesiosis; NA—not applicable; CT—Connecticut; MA—Massachusetts; MN—Minnesota; WI—Wisconsin; VT—Vermont; AZ—Arizona; NY—New York; NM—New Mexico; OK—Oklahoma; FL—Florida; RI—Rhode Island.

**Table 2:** Quantification of risk of transfusion transmitted babesiosis and assay development.

	Overview	Study Design	Major Finding	Reference
Efficacy of detection methods	Development of prototype EIA	Development of protype EIA using recombinant, immunodominant peptides BMN1-17 and MN-10.	69/72 (95.9%) IFA samples detected by EIA. 98/107 (91.5%) positive IgG blot samples detected using EIA. 53/63 (84.1%) positive IgM blot samples detected by EIA. All 12 PCR positive samples detected.	Houghton et al. Transfusion 2002 [36]
	Development of a real time PCR assay for detection of *B. microti*	Investigational study combining spiking experiments, probit analysis, and performance assessment using clinical sample panels.	Spiking experiment positive rate of detection: 445 copies/mL: 100%; 44.5 copies/mL: 97.5%; 4.45 copies/mL: 81%. The blinded probit analysis: detection rate: 95%: 12.92 parasites/2 mL; 50%: 1.52 parasites/2 mL of whole blood; Clinical samples: 13 of 21 samples were positive. Healthy donors: 0 of 48 positives.	Bloch et al. Transfusion 2013 [37]
	Development and validation of cobas *Babesia* assay (Roche diagnostics)	Evaluation of analytical performance of molecular assay (cobas *Babesia* assay, Roche diagnostics) targeting 4 major species of *Babesia* using individual and pooled samples Spiking experiments, cross-reactivity, and donor samples assessed to determine performance characteristics of the assay.	Limit of detection: *B. microti* 6.1 infected red blood cells (iRBC)/mL; *B. duncani* 50.2 iRBC/mL; *B. divergens* 26.1 iRBC/mL; *B. venatorum* 40.0 iRBC/mL. Specificity: ID-NAT: 99.999% (95% CI:99.996, 100); MP-NAT (6 donations): 100% (95% CI: 99.987, 100).	Stanley et al. Transfusion 2021 [38]
Parasite persistence in blood products	*Babesia* tolerance of storage conditions	*B. divergens* inoculated into blood bags containing leukoreduced red blood cells (RBCs) and stored at 4 °C for 0 to 31 days. Parasite viability assessed through interval sampling.	Viability maintained through 31 days of refrigerated storage despite altered morphology, reduction in parasitemia and lag to exponential growth.	Cursino-Santos et al. Transfusion 2014 [39]
Animal models for determining the risk of TTB.	Immunopathogenesis	6 Rhesus macaque monkeys were transfused with either hamster or monkey-passaged *B. microti*–infected red blood cells to simulate TTB	First detectable parasitemia 4 days in monkey-passaged cells (vs. 35 days in hamster passaged cells). Window period (detectable parasitemia by qPCR to detected antibody response): 10 to 17 days. Multilineage immune activation albeit not NK or Treg cells.	Gumber et al. Transfusion 2016 [40]
	Minimum infectious dose and kinetics of parasitemia	Murine model infected with different dilutions of *B. microti* parasitemic blood. Responses compared between immunocompetent and immunodeficient mice.	Peak parasitemia: 2 × 10^7^ pRBCs/mL at 2 to 3 weeks and 5 × 10^8^ pRBCs/mL at 6 weeks immunocompetent and immunodeficient, respectively. Chronic infection: fluctuating parasitemia in immunocompetent mice; high plateau parasitemia in immunodeficient mice. Minimum infectious dose: 100 parasitized RBCs in immunocompetent mice and 63 parasitized RBCs in immunodeficient mice; able to establish infection in all mice in respective cohorts.	Bakkour et al. Transfusion 2018 [35]

**Abbreviations:** IFA—indirect fluorescent antibody; EIA—enzyme immunoassay; PCR—polymerase chain reaction; TTB—transfusion-transmitted babesiosis; IDT—individual donor testing; MP-NAT—minipool nucleic acid testing.

**Table 3:** Transfusion-transmitted babesiosis: blood donor surveillance and quantification of transfusion-associated risk outside of the United States.

Study Design	Overview	Location (s)	Year (s)	Major Finding	Reference
Case report	53 y old female transfused for anemia secondary to gastrointestinal bleeding; found to be due to tumor of small intestine.	Ontario, Canada	1998	Parasites demonstrated on blood smear and diagnosis of *B. microti* infection confirmed by PCR. Donor implicated (smear-, PCR-, and IFA-positive). The donor had been camping in Cape Cod, Massachusetts (USA)	Kain, et al. Canadian Medical Association Journal 2001 [47]
Case report	40 y old male transfused for gastric bleeding; 1 month later the patient was investigated for fever and hemolysis.	Japan	1998–1999	Parasites demonstrated on blood smear and diagnosis of *B. microti* infection confirmed by PCR. Donor implicated.	Matsui et al. Rinsho Ketsueki 2000 [14]
Pilot serosurvey	Retrospective IFA screening for *B. divergens and B. microti* IgG antibodies.	North and East Tyrol, Austria	Not stated	Total of 988 blood donors screened (cut-off titer 128). 21/988 (2.1%) seroreactive for IgG antibodies against *B. divergens.* 5/988 (0.6%) reactive against *B. microti.*	Sonnleitner et al. Transfusion 2014 [48]
Tick surveillance to guide donor serosurvey	Passive surveillance of ticks used to identify regions for tick drag sampling. All ticks were tested for *B. microti* using PCR. Blood donations from selected sites (based on tick testing and near-endemic US regions) tested for antibody to *B. microti*; donors subjected to questionnaire about risk travel and possible tick exposure.	Southern Manitoba, Ontario, Quebec, New Brunswick, and Nova Scotia, Canada	2013	13,993/26,260 (53%) donors at the selected sites tested; none were positive for antibody to *B. microti*. 41% reported travel to the United States.	O’Brien et al. Transfusion 2016 [49]
Pilot serosurvey	Retrospective IFA screening of blood donor samples for *B. microti* antibodies.	Heilongjiang Province, China	2016	888 whole blood and 112 platelet donor samples (*n* = 1000); 13/1000 (1.3%) donors were seroreactive; 0.8% at a titer of 64 and 0.05% at titer of 128.	Bloch et al. Vox Sanguinis 2018 [50]
Surveillance study	NAT (TMA) screening of 50,752 blood samples and IFA screening of a subset of TMA-nonreactive samples (14,758).	Canadian regions close to US border, including British Columbia, Alberta, Saskatchewan, Manitoba, Ontario, Quebec, and Nova Scotia	2018	1/50,752 TMA-reactive; 4/14,758 antibody-positive.	Tonnetti et al. Transfusion 2018 [51]
Pilot serosurvey	Retrospective IFA screening of blood donor plasma samples for *B. microti* IgG antibodies; initially reactive samples were further tested for *B. microti* IgG and IgM by immunoblot and *B. microti* DNA by PCR.	New South Wales and Queensland, Australia	2012–2013	0 (0%) confirmed positive. 5 initial reactive donors failed to confirm on repeat/confirmatory testing.	Faddy et al. Transfusion 2019 [52]
Risk modelling study	Monte Carlo simulation used to estimate the number and proportion of *B. microti* infectious red blood cell units in Canada for three scenarios: base, localized incidence, and prevalence from donor data.	Canada	N/A	Expected NAT-positive donations per year (and clinically significant TTB): • Base scenario: 0.5 (0.08) (1 every 12.5 years). • Localized incidence scenario: 0.21(0.04) (about 1 every 25 years). • Donor study informed scenario: 4.6 (0.81)	O’Brien et al. Transfusion 2021 [53]

**Table 4:** Approaches to address the risk of TTB.

Approach	Strengths	Limitations
Risk-based deferral	Low cost Logistically simple	• Lack of specificity • High proportion of individuals are asymptomatic and parasitemia may be protracted • Most are unaware of past or active infection • Large number of reported cases of TTB in endemic areas
Peripheral blood smear	• Direct observation of parasites	• Not amenable to high-throughput donor screening • Low sensitivity • May lend itself to misdiagnosis, e.g., with *Plasmodium*
Serology	• Relatively low cost	• Poor correlation with active parasitemia risks intolerable rates of deferral in highly endemic areas • Limited cross-reactivity between *Babesia* species, such that other species may go undetected e.g., *B. duncani* • Variable performance of automated antibody tests in use that have largely been confined to the detection of *B. microti* antibodies • Rarity of selected species complicates validation of serological assays
Molecular methods	• Detectable RNA or DNA is a reasonable correlate of active parasitemia • Lower rates of reactivity than would be expected with serological testing, thus preventing high rates of donor deferral that would otherwise be encountered with serological testing • Central to blood donor screening policy in the US • Highly sensitive and specific, high-throughput licensed assays are available for donor screening; selected assays are also able to detect the major *Babesia* species using a single assay format • Enables donor reinstatement following deferral, i.e., after 2 years, if repeat testing is negative, individuals may be permitted to donate • Able to detect individuals in a pre-seroconversion window period	• Higher cost than serology • Imperfect correlate with active parasitemia, i.e., DNA/RNA may remain detectable following treatment or spontaneous resolution
Pathogen reduction	• FDA- and EU-approved photochemical inactivation technology is available for use in platelets and plasma; allowable as an alternative to molecular testing • Collateral benefits of pathogen reduction include efficacy against different classes of pathogens, including bacteria, thus addressing another major infectious risk to blood supply; also effective for prevention of transfusion-associated graft vs. host disease	• Absence of a licensed pathogen reduction technology for red blood cells and whole blood; TTB has not been ascribed to apheresis-collected platelets and plasma • High cost • Lower platelet yields as compared to standard platelet products
